# Pre- and Postoperative Gait After Proximal Medial Gastrocnemius Recession for Chronic Plantar Fasciitis: An Exploratory 3D Gait Analysis of 29 Patients

**DOI:** 10.1177/24730114251394010

**Published:** 2025-12-11

**Authors:** Martin Okelsrud Riiser, Espen Ingvald Bengtson, Sandra Linnea Klund-Hansen, Ingvild Koren Maalen-Johansen, Marius Molund

**Affiliations:** 1 Department of Foot and Ankle Surgery, Department of Orthopaedic Surgery, Østfold Hospital, Grålum, Norway; 2 Department of Foot and Ankle Surgery, Department of Orthopaedic Surgery, Oslo University Hospital, Norway; 3 University of Oslo, Faculty of Medicine, Norway; 4 Human Movement Scientist, Sunnaas Rehabilitation Hospital, Nesodden, Norway

**Keywords:** proximal medial gastrocnemius recession, 3-dimensional gait analysis, plantar fasciitis, ankle dorsiflexion

## Abstract

**Background:**

Plantar fasciitis is a prevalent foot condition, often resolving without surgery. However, a subset of patients experiences persistent symptoms beyond 12 months, necessitating interventions like proximal medial gastrocnemius recession (PMGR). PMGR is hypothesized to alleviate plantar fasciitis by increasing ankle dorsiflexion. The procedure’s effects on gait remain unclear.

**Methods:**

A subgroup of 29 patients with chronic plantar fasciitis, treated with PMGR and stretching as part of the Plantar Fasciitis Cohort Study, was selected for this preoperative and 3-month postoperative 3-dimensional gait analysis (3DGA) study. Eligibility criteria included symptoms persisting for more than 12 months, failure of conservative treatments, and confirmed gastrocnemius tightness. Gait analysis was performed using a 3D motion capture system. The primary outcome was maximal ankle dorsiflexion during stance. Secondary outcomes included other kinematic, kinetic, and tempo-spatial gait variables potentially influenced by PMGR, the Gait Deviation Index (GDI), and passive ankle dorsiflexion.

**Results:**

Maximal ankle dorsiflexion during stance showed no significant change postoperatively (13.5 degrees [12.2, 14.9] vs 14.3 degrees [13.2, 15.3], *P* = .21). Secondary outcomes, including gait parameters and extremity-specific GDI scores, remained within normal ranges and showed no clinically significant changes. Passive ankle dorsiflexion increased significantly postoperatively, yet this did not translate to detectable changes in gait patterns. Patients demonstrated no notable gait deviations compared with a normative population pre- or postsurgery.

**Conclusion:**

Findings suggest that gait patterns are relatively robust and that increased joint range of motion does not appear to affect gait mechanics 3 months postoperative based on a single-segment foot model. Further studies are needed to investigate these findings and to explore the biomechanical mechanisms underlying symptom improvement.

**Level of Evidence:**

Level IV, exploratory prospective cohort study.

## Graphical abstract

**Graphical Abstract:**
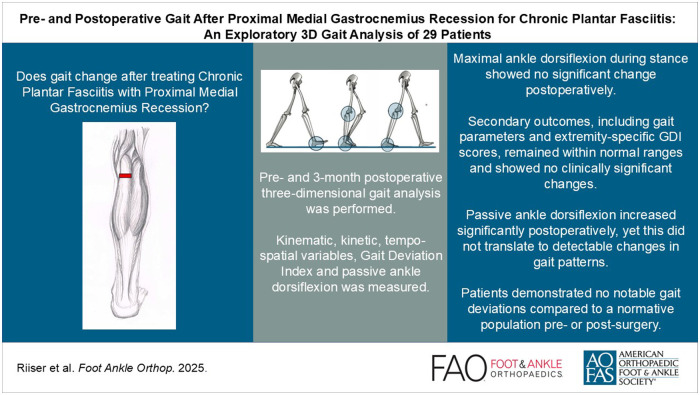
This is a visual representation of the abstract.

## Introduction

The pathophysiology of plantar fasciitis is multifactorial, with contributing factors including obesity, and overuse.^24^ Another notable factor is isolated tightness in the gastrocnemius muscle, which is implicated not only in plantar fasciitis but also in other foot and ankle conditions.^8,12,21,22^ This has led to the development of various gastrocnemius recession techniques as surgical treatment options.

Proximal medial gastrocnemius recession (PMGR), also known as Barouk procedure, is considered a safe and promising surgical option for treating chronic plantar fasciitis resistant to non-operative management.^23,30^ PMGR involves releasing the tightness of the gastrocnemius muscle by cutting the proximal tendon aponeurosis.^1^ The procedure has a low complication rate, and studies suggest that triceps surae muscle function remains intact at the 12-month and 6-year follow-ups.^19,25^ PMGR has been shown to increase passive ankle dorsiflexion.^3,19^ The relationship between increased ankle dorsiflexion and symptom improvement in plantar fasciitis remains unclear. It is hypothesized that gastrocnemius tightness alters gait and plantar pressure, contributing to plantar overload and plantar fasciitis. However, research on this topic is limited.

Given the limited evidence, there is a need for further research to determine whether PMGR (Barouk procedure) affects gait. The primary hypothesis of this study is that maximal ankle dorsiflexion during the stance phase increases after PMGR surgery. The study also aims to investigate whether other relevant kinematic, kinetic, or tempo-spatial variables change postsurgery, whether the gait of patients with plantar fasciitis differs from that of the normal population, and whether the previously observed increase in passive ankle dorsiflexion following PMGR can be reproduced.

## Methods

This study was a prospective cohort study, and the results presented focus specifically on gait analyses conducted within a subgroup of participants. The study was approved by the data protector officer at Østfold Hospital Trust and the Norwegian National Research Ethics Committees (REK 317597). It is registered at ClinicalTrials.gov (ref. NCT 05162144). Patient inclusion, treatment, and gait analysis for the subgroup took place between January 2022 and April 2024. A written consent was obtained from all participants.

### Participants

**Figure 1.**
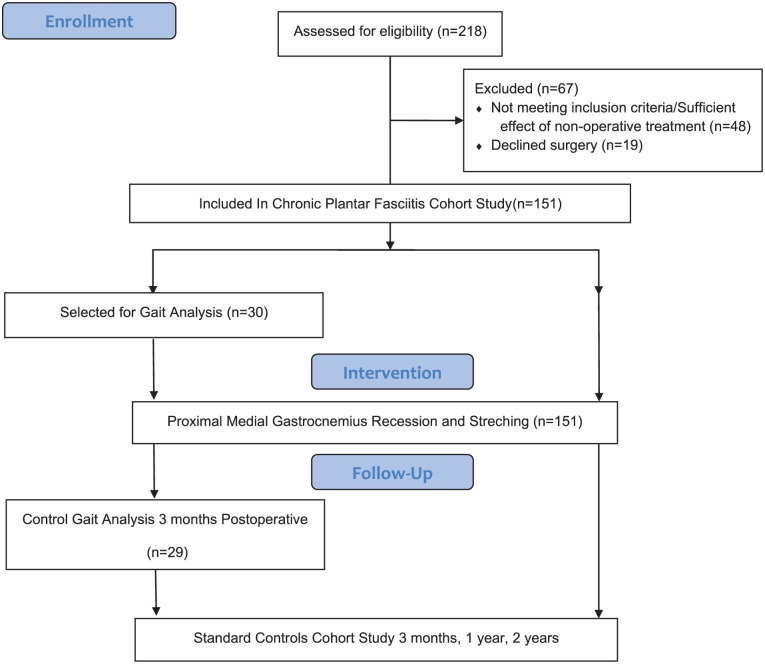
Flow chart describing inclusion and follow-up in the Chronic Plantar Fasciitis Cohort Study.

Patients with symptoms of plantar fasciitis referred to the orthopaedic department at Østfold Hospital Trust were evaluated for PMGR surgery and potential inclusion in the Plantar Fasciitis Cohort Study. A total of 151 patients meeting the inclusion criteria were enrolled in the study (Figure 1). Eligibility required patients to be 18-75 years old, have plantar fasciitis symptoms for >12 months, have failed conservative treatment with stretching exercises, and have a tight gastrocnemius muscle confirmed by Silfverskiöld test.^2,8^ Exclusion criteria were prior plantar fasciitis surgery, severe talocrural pathology, significant foot or ankle malalignment, poor peripheral circulation, chronic foot ulcers, a history of alcoholism or drug abuse, psychological or emotional issues affecting informed consent, inability to walk unaided, or inadequate proficiency in a Scandinavian or English language.

From the cohort of 151 patients, a convenience sample of 30 were selected for participation based on living proximity to the gait analysis laboratory at Sunnaas Rehabilitation Hospital, as well as the patients' willingness and availability to participate. Twenty-nine of the 30 patients completed both the baseline and 3-month 3DGA. Baseline data were obtained (age, sex, height, weight, BMI, presence of valgus/varus deformities, and duration of symptoms) before the preoperative 3DGA. In cases of bilateral plantar fasciitis, only the most affected foot underwent surgery before the 3-month postoperative 3DGA.

### Procedures

**Figure 2.**
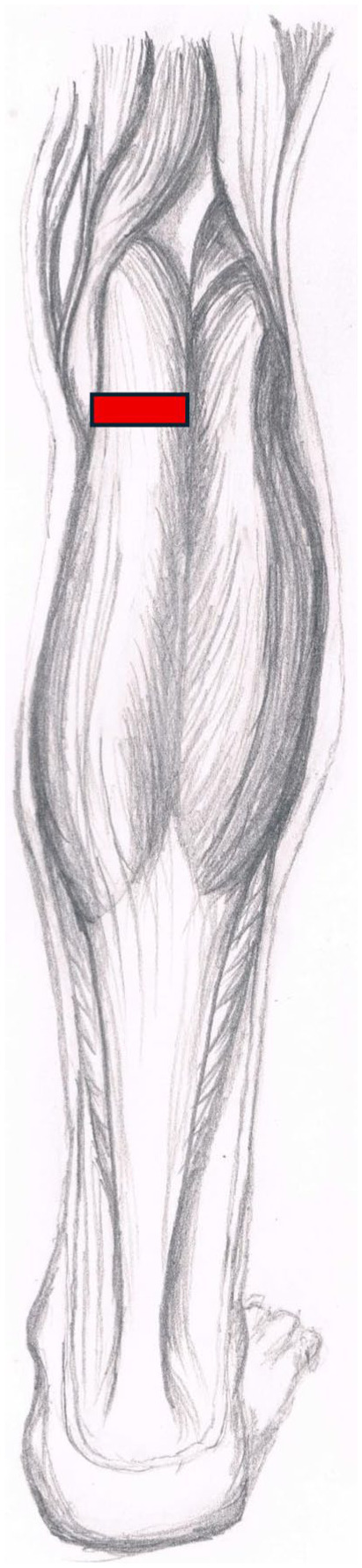
Level of proximal medial gastrocnemius recession (Courtesy of Maria Serafin).

All patients in the Cohort Study received PMGR surgery. The surgery was performed as described by Barouk (Figure 2).^1^ This procedure is usually performed under local anesthesia and involves cutting the tendon sheath/aponeurosis of the proximal medial gastrocnemius muscle horizontally and semi-circumferentially, through an approximately 4-cm incision, slightly inferior to the popliteal fossa. Only soft dressings were applied. Patients were instructed to fully weight-bear from the first postoperative day and to do daily stretching exercises of the calf for at least 3 months. Sutures were removed 2-3 weeks after surgery.

### Outcomes

**Figure 3.**
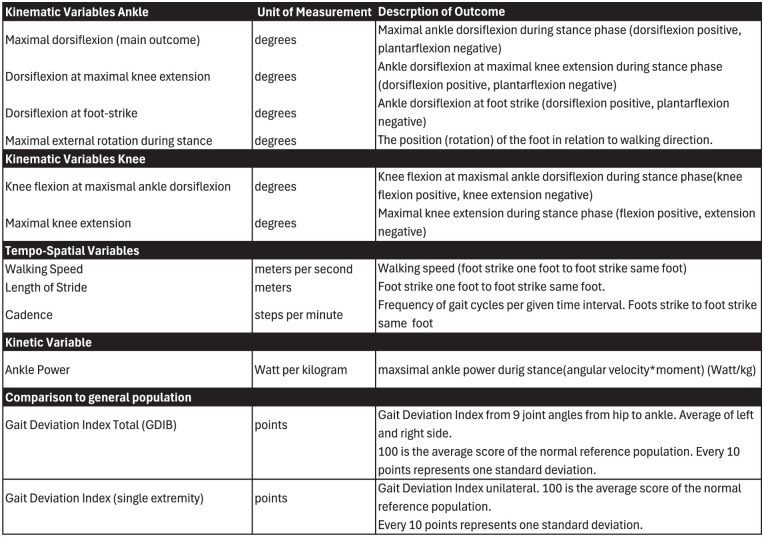
Three-Dimensional Gait Analysis Variables.

**Figure 4.**
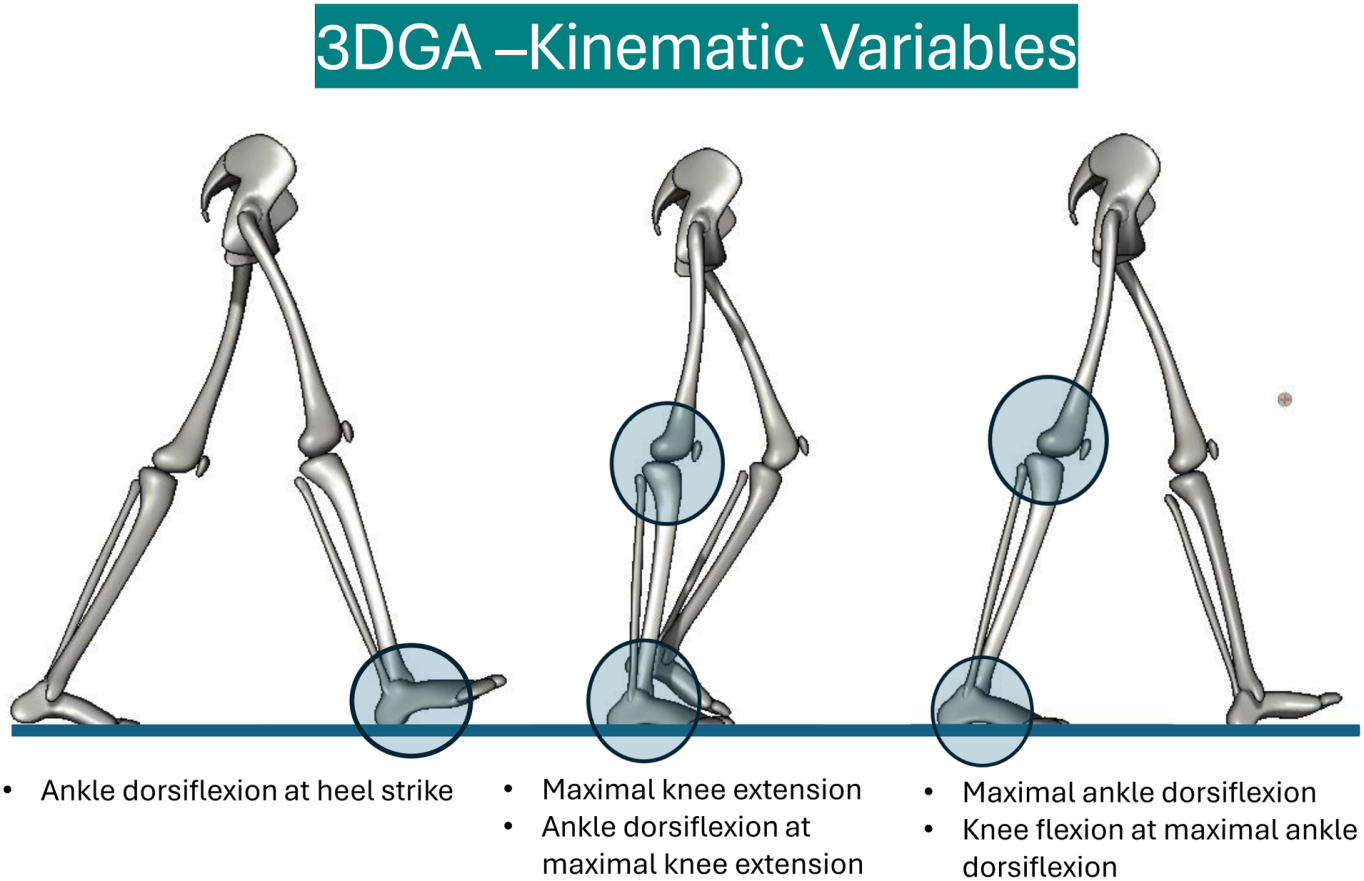
Three-Dimensional Gait Analysis – Kinematic Variables.

The main outcome of the gait analysis was the kinematic variable Maximal Ankle Dorsiflexion During Stance (degrees) (Figures 3 and 4). Treatment with PMGR surgery is based on the hypothesis that a tight gastrocnemius muscle affects plantar pressure because the tight gastrocnemius restricts ankle dorsiflexion in the stance phase. With our main outcome we wanted to test the hypothesis that PMGR surgery increases ankle dorsiflexion in the stance phase.

Secondary outcomes (Figures 3 and 4) were the kinematic variables ankle dorsiflexion at maximal knee extension, ankle dorsiflexion at foot strike, foot progression angle, knee extension at maximal ankle dorsiflexion, and maximal knee extension during stance. By adding these kinematic variables, we wanted to consider the more complex nature of gastrocnemius, soleus, and Achilles-complex function: the muscle complex affects movement of the knee because of its origin on the distal femur, and it affects several movements in the foot and ankle by surpassing both the ankle and the subtalar joint before it inserts on the calcaneus. Tightness of the gastrocnemius muscle may affect movement in both the ankle and the knee, and a potential restriction in the movement of one joint is therefore dependent on the position of the other.

Tempo-spatial variables were walking speed, stride length, and cadence. To measure a potential reduction of push-off power in the ankle due to surgery, we used the kinetic variable Maximum Ankle Power (watt/kg).

**Figure 5.**
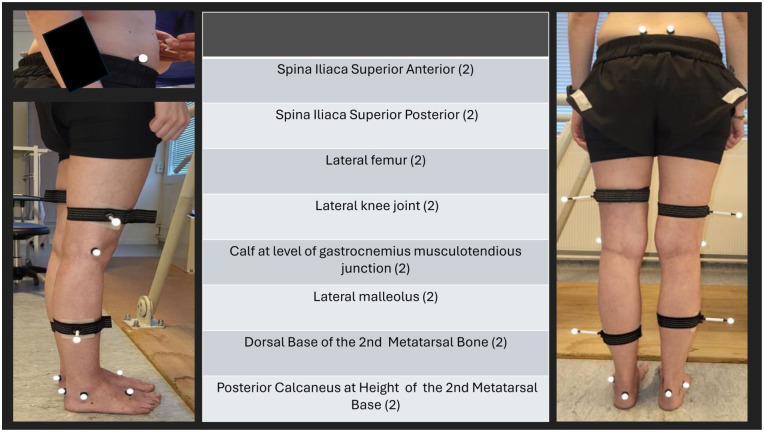
Placement of retroreflective markers (16).

The 3DGA was performed preoperatively and at 3 months postoperatively. It was carried out at the motion laboratory at Sunnaas Rehabilitation Hospital using a Vicon system of 2 AMTI force plates (AMTI Force & Motion, Watertown, MA) and 8 infrared cameras (Vicon Motion Systems, Oxford, United Kingdom). Sixteen retroreflective markers were placed on the lower extremities using the Vicon Plug-in-gait model (Vicon Motion Systems) (Figure 5). 3DGA is considered the most comprehensive gait analysis.^31^ The Plug-in-Gait model is a standardized biomechanical model for clinical gait analysis, based on the validated Newington-Helen Hayes model.^7^ Reliability of 3DGA is high, with the standard error of measurement reported to be <5 degrees (often less) for movements of the lower extremity.^18^ A general assumption for 3GDA is that adult walking patterns are considered to be present from the age of 13 years and are considered to be stable until late adulthood (up to the age of 70 years).^15,29^

The participants were asked to walk barefoot, with a self-selected comfortable speed on a 10-m walkway. The procedure was repeated until 3 representative trials of each leg was obtained. Tempo-spatial parameters and kinematic and kinetic variables were computed using Nexus software and a custom-made Python script pre- and postsurgery. All gait variables were exported automatically to Microsoft Excel with the Python script.

The Gait Deviation Index (GDI) was used to compare the participants gait to a general population.^28^ The GDI reflects a subjects’ overall gait pattern deviations compared to a reference population where a score of ≥100 is considered normal gait pattern, and 1 SD is 10 points below. The GDI uses data from across the gait cycle, focusing on 9 joint angles that are considered most clinically significant, including pelvis and hip angles in 3 planes, knee and ankle angles in the sagittal plane, and foot progression. It has been applied successfully to patients with a range of different conditions.^10,14,17^ GDI was measured individually for each leg and as an average of both legs. In the current study, a local reference material of 48 healthy adults were used in the calculations of GDI.^26^ Most relevant to this study is that the GDI has been shown to be an appropriate measure of changes in overall walking abilities following gastrocnemius fascia lengthening in children with cerebral palsy.^5^

The hypothesis that ankle dorsiflexion increases following PMGR surgery was also evaluated through passive ankle dorsiflexion measurements. A validated goniometer was used to measure passive ankle dorsiflexion with the knee extended, under a standardized plantar pressure of 50 N applied to the distal metatarsals through a dynamometer.^20^

### Sample Size Calculations and Statistical Analysis

The SD of peak ankle dorsiflexion during stance was reported to be in the range of 3.1-4.2 in a comparable study by Chimera et al.^4^ To be conservative, we used an SD of 5.0 to perform our sample size calculation. To our knowledge, a clinically important difference for peak ankle dorsiflexion during gait has not been established. For the purpose of this study, we considered 5 degrees to be clinically meaningful, based on previous reports of increased dorsiflexion following PMGR.^19^ Using a paired sample *t* test, and with power analysis of 80%, and a 5% level of significance, 16 patients were needed to prove a significant difference between maximal ankle dorsiflexion during stance before and after treatment. Because little data exist on this subject, we wanted to include 30 patients to avoid underpowering and compensate for potential loss to follow-up. The before and after data was examined for normal distribution using q-q plots and compared with paired *t* tests, and a *P* value less than .05 was considered significant. As patients only had received unilateral surgery at the time of the 3-month postoperative gait analysis, data from the non-operated leg could also be used as a control, using the same statistical methods. Baseline characteristics from patients completing both baseline and control gait analysis was compared to the remaining cohort with 2-sample *t* test or χ^2^ tests when appropriate. Median baseline characteristics between the gait analysis group and the GDI reference population were compared descriptively only, as individual-level data for the reference group were unavailable. Only patients completing pre- and postoperative 3DGA were included in final analysis. Given the exploratory nature of this study and the lack of prior data on the effect of PMGR on multiple gait variables, we chose not to apply a strict correction for multiplicity. Instead, we used an unadjusted *P* value threshold of .05 to identify trends that may warrant further investigation in larger confirmatory studies.

Statistical analyses were performed using Stata, version 18.0, Standard Edition (StataCorp, College Station, TX).

## Results

There were no significant differences in baseline demographic factors (age, sex, height, weight, and BMI) or the ratio of varus and valgus deformities between patients who underwent gait analysis and those who did not in our cohort of 151 patients. However, patients who underwent gait analysis had a significantly shorter symptom duration (Table 1A). When descriptively comparing baseline characteristics of patients who underwent gait analysis to the GDI reference population, the gait analysis group showed higher median age and weight. Height and gender distribution were comparable between groups (Table 1B).

**Table 1.** Baseline Data Gait Analysis.

A: Comparison Within Cohort (N = 151 patients)				
	Gait Analysis Group (n = 29)	Non–Gait Analysis Group (n = 122)	Mean Difference	*P* value
Age, y, mean (95% CI)	49.4 (45.2, 53.6)	48.1 (46.1, 50.1)	1.3 (−3.3, 5.8)	.58 ^a^
Weight, kg, mean (95% CI)	86.6 (81.6, 91.4)	85.2 (82.3, 88.1)	1.3 (−5.1, 7.7)	.67 ^a^
Height, cm, mean (95% CI)	171.3 (167.7, 174.8)	170.7 (169.1, 172.3)	0.6 (−3.2, 4.33)	.77 ^a^
Body mass index, mean (95% CI)	29.6 (27.8, 31.4)	29.2 (28.3, 30.1)	0.4 (−1.6, 2.4)	.69 ^a^
Symptom duration, mo, mean (95% CI)	28.2 (18.7, 37.7)	56.1 (44.3, 67.8)	−27.9 (−52.4, −3.3)	.03 ^a^
			ARD (IQR)	
Gender ratio: male/female, n	8:21	24:98	−0.08 (−0.25, 0.10)	.35 ^b^
Valgus deformity ratio: yes/no, n	3:26	20:102	−0.07 (−0.19, 0.06)	.42 ^b^
Varus deformity ratio: yes/no, n	5:24	26:96	−0.05 (−0.20, 0.10)	.63^b^
B: Descriptive Comparison: Gait Analysis Group vs GDI Reference Population^c^				
	Gait Analysis Group (n = 29)	Reference Population GDI (n = 48)		
Age, y, median (IQR)	47(24-72)	40.5 (23-62)		
Gender ratio, male/female, n (% male)	8:21 (38.1)	10:38 (26.3)		
Weight, kg, median (IQR)	86 (50-115)	70.9 (53.7-117)		
Height, cm, median (IQR)	170 (158-199)	173 (157-194)		

Abbreviations: ARD, absolute risk difference; GDI, Gait Deviation Index.

a Two-sample *t* test.

b χ^2^ test.

c No individual data from reference population were available for statistical comparison.

Results of the 3DGA are presented in Table 2. Individual data for each of the 29 patients who completed both baseline and 3-month gait analysis are presented in Appendix A.

**Table 2.** Results Gait Analysis.

Outcome	Baseline	3 mo Postop	Mean Difference	*P* Value
Kinematic variables: Ankle				
Main outcome: Maximal ankle dorsiflexion during stance (degrees)				
Operated leg	13.5 (12.2, 14.9)	14.3 (13.2, 15.3)	0.8 (−0.4, 1.9)	.21
Non-operated leg	13.4 (12.1, 14-8)	14.3 (13.0, 15.6)	0.9 (−0.4, 2.1)	.16
Mean difference	0.1 (−1.3, 1.4)	0.0 (−1.0, 0.9)		
*P* value	.90	.93		
Dorsiflexion at maximal knee extension (degrees)				
Operated leg	8.3 (6.7, 10.0)	7.3 (5.3, 9.3)	−1.0 (−3.1, 0.9)	.29
Non-operated leg	7.1 (5.1, 9.1)	7.0 (4.6, 9.4)	−0.1 (−2.5, 2.4)	.96
Mean difference	1.2 (−0.9, 3.4)	0.3 (−1.8, 2.2)		
*P* value	.25	.81		
Dorsiflexion at foot-strike (degrees)				
Operated leg	−5.1 (−7.0, −3.2)	−3.7 (−5.1, −2.7)	1.4 (0.0, 2.8)	.05
Non-operated leg	−3.8 (−5.0, −2.7)	−3.7 (−5.0, −2.4)	0.1 (−1.2, 1.4)	.86
Mean difference	−1.3 (−3.0, 0.5)	0.0 (−0.8, 0.8)		
*P* value	.15	.95		
Maximal external rotation during stance (degrees)				
Operated leg	−8.0 (−10.2, −5.7)	−7.6 (−10.0, −5.1)	0.4 (−0.6, 1.5)	.45
Non-operated leg	−8.8 (−10.6, −7.0)	−8.0 (−10.0, −6.1)	0.8 (−0.4, 2.0)	.18
Mean difference	0.8 (−0.6, 2.2)	0.4 (−1.5, 2.3)		
*P* value	.25	.66		
Kinematic variables knee				
Knee flexion at maximal ankle dorsiflexion (degrees)				
Operated leg	5.3 (3.0, 7.6)	5.1 (2.7, 7.6)	−0.2 (−2.1, 1.8)	.88
Non-operated leg	4.1 (1.9, 6.2)	4.8 (2.5, 7.2)	0.7 (−1.1, 2.6)	.41
Mean difference	1.2 (−0.5, 2.9)	0.3 (−1.6, 2.3)		
*P* value	.15	.74		
Maximal knee extension (degrees)				
Operated leg	−0.3 (−2.1, 1.4)	−0.5 (−2.4, 1.5)	−0.2 (−2.1, 1.8)	.88
Non-operated leg	−1.0 (−3.1, 1.0)	−0.8 (−2.8, 1.3)	0.2 (−1.8, 2.4)	.79
Mean difference	0.7 (−0.8, 2.2)	0.3 (−1.3, 1.9)		
*P* value	.34	.71		
Tempo-spatial variables				
Walking speed (m/s)				
Operated leg	1.20 (1.13, 1.27)	1.21 (1.13, 1.28)	0.01 (−0.05, 0.06)	.85
Non-operated leg	1.20 (1.13, 1.27)	1.21 (1.13, 1.28)	0.01 (−0.05, 0.06)	.75
Mean difference	0.0 (−0.01, 0.01)	0.0 (−0.01, 0.01)		
*P* value	.73	.51		
Length of stride, m				
Operated leg	0.63 (0.60, 0.65)	0.63 (0.60, 0.66)	0.01 (−0.01, 0.03)	.32
Non-operated leg	0.63 (0.61, 0.66)	0.64 (0.62, 0.67)	0.01 (−0.01, 0.02)	.42
Mean difference	−0.01 (−0.02, 0.00)	−0.01 (−0.02, 0.01)		
*P* value	.03	.39		
Cadence (steps/min)				
Operated leg	114 (110, 119)	113 (108, 118)	−1 (−5, 3)	.63
Non-operated leg	114 (110, 118)	114 (109, 118)	0 (−4, 3)	.62
Mean difference	0 (−1, 0)	1 (−1, 0)		
*P* value	.46	.35		
Kinetic variable				
Maximum ankle power (W/kg)				
Operated leg	3.6 (3.3, 3.9)	3.7 (3.3, 4.1)	0.1 (−0.2, 0.5)	.50
Non-operated leg	3.9 (3.5, 4.2)	4.0 (3.6, 4.4)	0.1 (−0.2, 0.4)	.51
Mean difference	−0.3 (−0.5, 0.0)	−0.3 (−0.5, 0.0)		
*P* value	.03	.053		
Comparison to normal population				
Gait Deviation Index Total: Both lower extremities	94.0 (90.2, 97.9)	90.3 (86.6, 94.1)	−3.7 (−7.2, −0.2)	.04
Gait Deviation Index per extremity				
Operated leg	94.3 (90.2, 98.3)	91.5 (87.7, 95.4)	−2.7 (−6.8, −1.3)	.18
Non-operated leg	95.0 (91.3, 98.6)	91.3 (87.3, 95.4)	−3.6 (−7.3, 0.0)	.05
Mean difference	0.7 (−2.9, 1.5)	0.2 (−2.3, 2.8)		
*P* value	.51	.87		

Values are presented as means with 95% Confidence Intervals.

For the main outcome, maximal ankle dorsiflexion during stance, no significant differences were found in the operated extremity between baseline and the 3-month follow-up (13.5 degrees [12.2, 14.9] vs 14.3 [13.2, 15.3] degrees (*P* = .21). The non-operated extremity also showed no significant changes from baseline to the 3-month follow-up. Furthermore, there were no differences between the operated and non-operated extremities at either time point.

Similarly, no significant differences were found between the baseline and the 3-month follow-up for most secondary outcomes in the 3DGA, whether for the operated extremity, non-operated extremity, or between the 2 extremities. This includes ankle dorsiflexion at maximal knee extension, ankle dorsiflexion at foot strike, foot progression angle, knee extension at maximal ankle dorsiflexion, maximal knee extension during stance, walking speed, stride length, cadence, and maximal ankle power.

The GDI showed a significant reduction in the total index score from baseline to the 3-month postoperative follow-up. However, no significant differences were observed in the GDI of individual extremities when comparing the baseline and 3-month follow-up, nor between the operated and non-operated extremities.

Measurements of passive ankle dorsiflexion (Table 3) revealed a significant increase in movement in the operated extremity from baseline to the three-month follow-up (−1.8 [−3.6, 0.1] degrees vs 4.3 [2.3, 6.4] degrees, *P* < .01). Conversely, the non-operated extremity showed no change in ankle dorsiflexion (0.0 [−2.3, 1.7] degrees vs −0.3 [−2.1, 2.0] degrees, *P* = .78).

**Table 3.** Passive Ankle Dorsiflexion Measurements.

	Maximal Passive Ankle Dorsiflexion of Extended Knee, degrees^a^			
Outcome	Baseline	3 mo Postop	Mean Difference	*P* Value
Operated leg	−1.8 (−3.6, 0.1)	4.3 (2.3, 6.4)	6.1 (4.2, 8.0)	<.01
Non-operated leg	0.0 (−2.3, 1.7)	−0.3 (−2.1, 2.0)	−0.3 (−2.7, 1.7)	.78
Mean difference	−1.8 (−4.2, 0.8)	4.6 (2.7, 6.9)		
*P* value	.17	<.01		

a Measured with goniometric device.

## Discussion

Our study found no significant changes in gait following PMGR surgery and stretching for chronic plantar fasciitis, based on pre- and postoperative 3D gait analysis at 3 months. No differences were observed in maximal ankle dorsiflexion during the stance phase, or in other kinematic variables of the knee and ankle (Table 2). Furthermore, maximal ankle power and spatiotemporal gait parameters such as stride length, walking speed and cadence remained unchanged. Taken together, within this timeframe and analytic model, we did not detect measurable changes in gait after PMGR.

Baseline body weight and age were higher in the study population than in the healthy reference group. A higher body weight could be anticipated in our study population, as it represents a well-established risk factor for plantar fasciitis.^24^ Preoperatively, GDI scores for both the total GDI and each extremity separately were within 10 points of the normal reference score. This indicates that patients with chronic plantar fasciitis do not exhibit a measurably altered gait compared with a normal population. From baseline to the 3-month follow-up, there was a statistically significant reduction in the total GDI score, decreasing from 94.0 (90.2, 97.9) to 90.3 (86.6, 94.1) (*P* = .04). However, this decrease was not observed when analysing each extremity individually. Notably, all measurements remained within the range considered normal for GDI.^28^ The Minimal Clinically Important Difference (MCID) for GDI has not yet been established for adult populations; however, the minimal detectable change is reported to be about 7.5 points for the non-paretic leg of adults post-stroke.^6^ Thereby, it is uncertain if a reduction of 3.7 points is due to measurement errors, and it is unlikely to be clinically significant.

Our study found a significant increase in passive ankle dorsiflexion with extended knee when comparing baseline measurements to the 3-month postoperative follow-up. These results align with findings from previous clinical studies that reported increased ankle dorsiflexion following gastrocnemius recession procedures, and suggest that lengthening the gastrocnemius increases ankle motion.^9,11,19^ However, in our study, and in contrary to our hypothesis, this did not translate to an increase in ankle dorsiflexion during gait. This suggests that gait patterns are robust because of neuromuscular habituation, indicating that an increased range of motion in a joint does not necessarily result in immediate or direct changes to gait patterns.

To our knowledge, this is the first study to evaluate the effects of PMGR (Barouk procedure) on gait. Previous gait analysis studies of gastrocnemius recession have primarily involved patients with cerebral palsy and equinus deformities.^13,16^ Although these studies consistently report increased postoperative ankle dorsiflexion, they are not directly comparable to ours because of differences in study populations and the frequent use of more extensive distal procedures (eg, Strayer, Baumann, or Achilles tendon lengthening). The only comparable study in a similar patient population was published by Chimera et al^4^ in 2012, in which 6 patients underwent a Strayer gastrocnemius recession. Despite differences in surgical technique, the findings of both studies are consistent in suggesting that gait mechanics remain largely unaltered following surgery. In contrast to Chimera's study, which identified an abnormal gait pattern in patients with plantar fasciitis, our study found no notable gait disturbances in this population when compared to a reference group, as assessed using the GDI.

The main strength of this study is its larger sample size compared to the only comparable study currently available. Additionally, as the baseline characteristics align closely with those of the broader cohort of 151 patients with chronic plantar fasciitis, our findings may be applicable to a wider population of patients with this condition. Limitations are the lack of a control group that did not receive surgery. We have tried to compensate for this by comparing our study group’s GDI to a healthy reference population. We also compared outcomes with the non-operated extremity. The latter approach is problematic, as both limbs are governed by the same gait pattern and therefore cannot be considered independent. Moreover, the contralateral foot may present with a different level of symptoms, or none at all. Nevertheless, demonstrating that the results of the non-operated limb remained stable supports the reproducibility of our analyses across time points. Additionally, because the gait-analysis subgroup had a shorter symptom duration than the remainder of the PMGR cohort (Table 1A), selection effects may limit generalizability of the gait findings. We have not compared results from patients with uni- or bilateral plantar fasciitis. It can also be argued that changes in gait might appear at a later stage postoperatively; however, based on immediate effects seen on ankle dorsiflexion intraoperatively, and limited resources for multiple 3DGAs, we concluded that 3 months would be the most reasonable time for follow-up. Future studies should also focus on long-term results that may capture potential neuromuscular adaptations at a later postoperative stage.

It is important to note that the complex motion of the human foot and ankle cannot yet be fully replicated in computer models using current technology. Although 3DGA is widely employed not only for patients with severe motion impairments but also for examining gait in various populations with foot and ankle pathologies,^32^ limitations remain. Many studies use multisegmental foot models to capture intricate movements within the foot, especially when studying gait after ankle arthroplasty.^20^ However, such models were not accessible for our study. Additionally, we focused primarily on ankle movement, which we considered to be most affected by PMGR. Consequently, certain gait changes may not have been captured by our model. The quality of the study could also have been improved if we had been able to compare time-series joint angle data using statistical parametric mapping to understand kinematic regions that may differ pre- and postoperatively. We recommend the application of a multisegmental foot model and comparing of time-series joint angle data in future studies. Changes in plantar pressure after surgery was also left out of our study protocol for the same reasons. However, the senior author of this study has previously evaluated potential changes in plantar pressure in patients who underwent PMGR surgery. At 12 months postsurgery, he observed an increase in peak heel and forefoot pressure, presumably because of the resolution of pain.^20^

This study has not provided definitive answers regarding the pathophysiology linking plantar fasciitis to a tight gastrocnemius muscle. Although our study and others examining the effects of PMGR have observed an increase in passive ankle dorsiflexion, this improvement has not been shown to translate into detectable changes in gait patterns.^19,27^ Consequently, we must speculate that the alleviation of plantar fasciitis symptoms following PMGR surgery may be attributed to 1 or more of the following: gait changes that are undetectable using our model, alterations in plantar pressure, or internal biomechanical changes within the foot that do not manifest as observable changes in gait.

## Conclusion

Our study concludes that 3D gait analysis shows no changes in gait patterns between baseline and 3-month postoperative evaluations in patients with chronic plantar fasciitis treated with PMGR and stretching. Although passive ankle dorsiflexion is significantly increased, this improvement in range of motion does not result in observable changes in gait patterns. Furthermore, patients with chronic plantar fasciitis exhibit no notable gait deviations compared to a reference population, and this remains clinically unchanged following PMGR surgery and stretching. At 3 months, using a single-segment foot model, PMGR did not measurably alter gait metrics. However, because of the exploratory nature of this study and analytic limitations (eg, lack of multisegment modeling and time-series analyses), more confirmatory studies with larger patient series and longer follow-up time are needed to make more definite conclusions.

## Supplemental Material

sj-pdf-1-fao-10.1177_24730114251394010 – Supplemental material for Pre- and Postoperative Gait After Proximal Medial Gastrocnemius Recession for Chronic Plantar Fasciitis: An Exploratory 3D Gait Analysis of 29 Patients Supplemental material, sj-pdf-1-fao-10.1177_24730114251394010 for Pre- and Postoperative Gait After Proximal Medial Gastrocnemius Recession for Chronic Plantar Fasciitis: An Exploratory 3D Gait Analysis of 29 Patients by Martin Okelsrud Riiser, Espen Ingvald Bengtson, Sandra Linnea Klund-Hansen, Ingvild Koren Maalen-Johansen and Marius Molund in Foot & Ankle Orthopaedics

## Appendix A

**Table:** 3-Dimensional Gait Analysis – Individual Data.

		Maximal Ankle Dorsiflexion During Stance (degrees)				Ankle Dorsiflexion at Maximal Knee extension (degrees)				Ankle dorsiflexion at foot-strike (degrees)				Maximal external rotation during stance (degrees)				Knee flexion at maximal ankle dorsiflexion (degrees)				Maximal knee extension (degrees)					Walking Speed (m/s)				Length of Stride (m)				Cadence (steps per min)				Ankle Power (W/kg)				Gait Deviation Index		Gait Deviation Index (single extremity)			
		Operated Extremity		Non-operated Extremity		Operated Extremity		Non-operated Extremity		Operated Extremity		Non-operated Extremity		Operated Extremity		Non-operated Extremity		Operated Extremity		Non-operated Extremity		Operated Extremity			Non-operated Extremity		Operated Extremity		Non-operated Extremity		Operated Extremity		Non-operated Extremity		Operated Extremity		Non-operated Extremity		Operated Extremity		Non-operated Extremity				Operated Extremity		Non-operated Extremity	
ID	Bilat	Baseline	3-months	Baseline	3-months	Baseline	3-months	Baseline	3-months	Baseline	3-months	Baseline	3-months	Baseline	3-months	Baseline	3-months	Baseline	3-months	Baseline	3-months	Baseline	3-months		Baseline	3-months	Baseline	3-months	Baseline	3-months	Baseline	3-months	Baseline	3-months	Baseline	3-months	Baseline	3-months	Baseline	3-months	Baseline	3-months	Baseline	3 months	Baseline	3 months	Baseline	3 months
1	No	12.71	15.88	15.43	18.08	−3.71	5.03	−0.27	12.10	−3.71	−5.40	−2.69	−3.16	−14.84	−12.06	−8.45	−9.89	9.34	6.79	9.41	13.91	0.20	2.01		−3.17	8.05	0.92	1.23	0.93	1.24	0.55	0.61	0.54	0.63	100.77	119.53	102.69	119.09	2.37	4.84	2.68	4.86	95.94	91.75	98.79	93.39	93.08	90.09
2	Yes	10.95	12.26	6.29	11.05	9.00	8.97	1.79	8.07	−3.58	−0.64	−8.19	−1.42	−8.28	−8.07	−14.76	−14.58	0.44	−0.77	−5.04	−0.13	−4.41	−3.61		−10.12	−3.88	1.38	1.42	1.38	1.43	0.72	0.71	0.70	0.74	116.92	119.52	118.66	118.54	3.65	3.84	3.25	4.24	95.41	85.33	98.71	85.21	92.11	85.44
3	No	23.28	16.72	20.40	18.28	−0.52	−3.49	13.24	−4.75	−0.17	−1.86	−2.70	−2.27	−11.96	−10.74	−9.34	−9.81	23.09	15.77	19.40	17.28	12.17	7.10		12.46	7.26	1.03	1.23	1.02	1.19	0.49	0.51	0.54	0.55	120.41	135.00	118.82	133.20	3.54	4.09	3.90	4.71	79.75	88.14	78.92	90.22	80.57	86.05
4	No	17.73	18.97	16.06	15.21	6.62	3.09	4.45	3.99	−1.40	0.66	−2.63	−1.47	−13.80	−8.64	−14.85	−14.46	8.65	7.92	5.01	0.95	−0.12	−5.51		−3.74	−7.34	1.08	1.13	1.07	1.16	0.58	0.58	0.58	0.59	111.11	115.05	110.94	117.69	3.79	3.38	3.42	3.74	101.93	92.96	108.29	97.06	95.58	88.85
5	No	19.08	14.85	18.76	14.83	10.72	−4.83	2.72	5.75	0.77	−3.80	−2.44	−4.67	−11.77	−9.86	−3.49	−5.40	4.39	−4.26	5.27	−2.15	−1.21	−10.15		1.89	−6.55	0.99	1.04	0.99	1.02	0.59	0.62	0.62	0.63	97.27	98.98	97.54	97.43	2.32	3.13	2.71	2.59	75.80	75.17	76.43	73.75	75.18	76.59
6	No	10.89	8.92	12.74	10.31	9.61	6.36	9.37	6.61	−8.90	−9.42	−4.94	−7.90	−11.12	−17.38	−13.54	−14.05	9.10	3.70	3.58	3.40	5.20	0.55		−2.78	−1.76	1.05	1.07	1.04	1.10	0.53	0.57	0.52	0.58	120.67	111.11	117.79	112.92	2.29	2.50	2.72	2.78	81.61	75.58	74.01	76.21	89.21	74.95
7	Yes	13.43	10.32	11.02	10.55	11.48	7.51	8.32	5.78	−17.68	−6.64	−3.81	−2.48	−3.18	0.21	−6.50	−7.47	2.05	−1.02	0.80	−1.44	−0.40	−6.00		−2.64	−7.02	1.43	1.58	1.42	1.60	0.66	0.67	0.66	0.73	128.71	134.71	128.86	135.15	4.42	4.67	4.76	5.28	113.58	105.14	112.09	106.16	115.06	104.12
8	No	16.54	12.82	17.25	14.08	13.61	10.42	14.06	12.59	−1.92	−6.21	−3.06	−2.19	−5.15	−0.28	−9.55	−7.00	4.81	2.22	3.64	5.08	2.34	−0.06		0.28	2.82	0.89	0.93	0.91	0.92	0.55	0.58	0.54	0.54	96.74	99.63	99.80	98.05	2.43	2.35	3.04	2.91	92.49	94.51	96.48	103.90	88.49	85.11
9	Yes	15.78	12.19	12.83	12.72	12.71	8.74	7.41	8.04	1.44	−1.23	−3.47	−1.30	−20.46	−18.14	−19.52	−17.44	5.60	1.34	3.84	0.69	1.47	−3.83		−0.27	−4.39	0.89	1.03	0.91	1.03	0.55	0.61	0.59	0.59	95.14	103.26	96.02	103.71	2.05	2.36	2.32	3.02	90.45	96.89	90.23	96.22	90.66	97.56
10	Yes	13.48	14.02	14.54	14.07	11.75	12.49	13.46	11.58	−4.10	−3.13	−0.19	−2.18	−23.24	−25.39	−16.44	−18.70	1.75	5.63	5.72	1.67	−0.53	2.77		2.89	−1.69	1.25	1.26	1.24	1.24	0.65	0.65	0.62	0.63	117.79	118.18	116.75	116.15	3.93	4.38	4.00	4.74	109.57	109.30	112.65	102.38	106.47	116.20
11	Yes	16.10	13.93	14.40	10.08	15.91	10.17	13.22	9.38	−3.63	−6.91	−3.93	−7.56	−8.40	−8.08	−6.77	−8.51	6.90	−0.32	8.47	−1.81	6.68	−2.61		7.24	−3.34	1.16	1.19	1.17	1.19	0.63	0.65	0.63	0.63	111.13	111.66	111.71	111.11	3.95	4.34	3.91	4.14	88.21	87.88	87.56	85.71	88.85	90.06
12	Yes	12.66	17.10	12.67	20.88	8.60	5.47	9.09	13.69	3.44	2.10	5.15	−1.71	−7.97	−5.45	−10.20	−12.37	2.00	9.34	2.97	13.38	−3.23	5.27		−2.45	3.27	1.38	0.98	1.35	0.97	0.67	0.64	0.68	0.64	121.72	92.83	120.23	93.75	3.90	0.69	3.30	2.18	93.50	77.65	92.58	92.81	94.42	93.71
13	Yes	12.66	13.00	3.97	10.24	9.38	9.46	1.62	8.76	−4.68	−4.61	−8.97	−2.60	−12.67	−10.93	−12.13	−8.87	2.48	4.56	−6.50	−2.96	−0.37	0.73		−11.10	−4.84	1.06	1.11	1.06	1.08	0.56	0.63	0.61	0.63	107.63	104.11	109.83	104.35	4.27	4.43	2.87	3.39	83.45	86.45	81.42	108.75	85.47	96.22
14	Yes	7.65	14.86	17.46	19.08	3.48	9.60	12.09	13.44	−6.31	−4.01	−3.27	−2.22	−8.07	−11.54	−8.70	−6.76	−0.82	−0.02	6.97	7.82	−6.83	−5.10		2.50	2.24	1.35	1.31	1.34	1.31	0.78	0.78	0.80	0.78	101.75	99.43	101.35	101.18	3.70	4.22	4.05	4.16	91.71	97.24	93.95	94.10	89.46	100.37
15	Yes	13.85	16.52	11.86	12.47	5.52	5.86	0.91	−5.47	−6.90	−7.17	−5.82	−10.10	−8.97	−6.56	−14.86	−11.73	1.59	1.52	0.01	−2.06	−4.74	−5.52		−6.23	−11.33	1.24	1.16	1.23	1.15	0.59	0.56	0.59	0.58	126.87	120.65	124.53	121.60	4.50	4.65	4.97	4.74	94.50	78.44	94.65	78.67	94.35	78.19
16	Yes	11.49	15.12	13.72	11.57	9.98	12.81	9.80	3.47	−11.92	−3.65	−2.62	−3.54	−11.36	−18.47	−12.71	−8.37	4.69	8.97	6.09	5.61	2.41	5.77		3.12	2.55	1.05	1.05	1.06	1.05	0.63	0.65	0.67	0.67	97.33	94.82	97.57	95.65	3.15	3.11	3.10	2.60	105.18	97.17	106.37	91.84	104.00	102.49
17	Yes	13.99	17.50	15.51	19.66	2.47	2.10	−3.06	−0.80	−7.14	−3.04	−2.74	0.19	−0.69	0.38	−5.85	−1.76	2.10	15.07	8.98	17.54	−3.77	10.21		0.18	7.66	1.07	0.74	1.08	0.78	0.53	0.38	0.53	0.53	120.79	95.51	122.51	101.94	3.68	2.83	3.81	1.52	96.75	75.42	96.78	77.63	96.71	73.20
18	No	13.31	17.50	15.26	18.45	10.00	−6.94	8.79	−9.54	−14.59	−5.03	−6.36	−7.23	−11.86	−12.01	−12.12	−3.74	4.08	3.64	3.98	6.12	−1.01	−5.57		−3.57	−3.56	1.33	1.27	1.33	1.29	0.61	0.62	0.63	0.63	129.26	123.44	128.98	123.92	4.32	5.09	6.01	5.71	97.94	89.31	93.47	87.44	102.40	91.18
19	No	8.05	12.58	9.73	12.52	4.25	8.12	5.69	8.45	−3.35	−0.44	−1.89	−0.51	−3.44	−3.19	−4.65	−5.73	1.82	9.09	0.19	10.69	−2.96	1.69		−5.31	4.03	1.10	1.10	1.08	1.12	0.60	0.62	0.59	0.59	110.26	109.44	109.63	111.16	3.76	3.94	3.84	4.56	107.28	87.90	104.16	87.17	110.40	88.63
20	Yes	13.03	14.46	11.47	11.59	9.99	7.21	7.47	9.03	−2.78	−0.61	−0.76	−0.93	−5.45	−6.22	−5.10	−7.42	5.90	9.71	−0.71	2.06	0.83	4.56		−4.99	−3.09	1.36	1.21	1.34	1.19	0.63	0.59	0.66	0.61	126.27	121.03	124.60	119.36	4.28	3.43	4.77	4.28	93.72	91.79	94.76	92.61	92.67	90.95
21	No	14.35	15.24	17.01	19.34	10.23	11.64	−7.59	−3.63	−6.82	−3.82	−6.91	−2.16	−1.00	−1.87	−5.88	−9.36	9.61	11.19	12.84	15.78	2.65	6.44		3.17	8.51	1.49	1.45	1.47	1.43	0.66	0.63	0.64	0.61	136.40	139.16	136.69	136.05	5.71	5.41	5.10	5.34	84.69	93.67	83.35	97.08	86.02	90.25
22	No	11.30	11.35	14.38	12.88	7.17	7.61	9.25	8.15	−4.81	−4.54	−6.45	−8.82	−1.33	−0.01	−1.15	−1.99	−0.20	−5.10	−0.88	0.99	−5.16	−9.35		−5.31	−4.04	1.40	1.33	1.39	1.34	0.68	0.68	0.72	0.70	119.50	114.77	117.57	115.63	3.64	3.42	4.78	4.38	93.58	84.35	91.83	81.42	95.33	87.28
23	Yes	11.15	12.03	10.40	8.58	9.36	9.98	8.61	7.79	−7.57	−4.19	−4.35	−3.73	−0.19	−1.86	−3.26	−5.77	−3.49	−0.71	−2.31	−2.45	−6.48	−3.21		−4.94	−9.16	1.46	1.51	1.47	1.50	0.67	0.70	0.67	0.68	131.36	130.74	131.34	130.88	4.72	4.59	4.88	4.04	110.39	94.04	111.35	95.26	109.43	92.81
24	No	8.34	8.88	14.83	12.17	5.86	6.82	10.46	10.70	−7.90	−7.10	−5.11	−5.26	−6.85	−5.36	−4.91	−2.73	3.39	−0.84	2.90	2.69	−3.63	−4.80		−0.93	0.36	1.09	1.35	1.10	1.37	0.62	0.69	0.63	0.66	105.35	119.67	105.68	121.72	2.71	4.58	3.88	4.48	102.71	102.38	99.17	101.02	106.24	103.73
25	Yes	17.34	19.18	15.98	18.67	12.78	16.71	13.57	16.04	0.12	2.53	−1.00	1.84	1.05	−0.75	−1.84	−6.25	17.93	8.82	15.82	9.85	10.47	5.20		12.52	5.65	1.15	1.22	1.14	1.24	0.58	0.64	0.58	0.64	117.14	115.84	117.70	116.60	3.00	2.92	3.63	4.27	80.70	77.78	78.98	78.59	82.41	76.95
26	No	8.89	11.92	10.50	13.32	3.33	5.72	8.10	11.17	−0.59	1.01	−4.22	−1.07	−8.41	−4.53	−7.94	−1.99	19.06	22.21	4.40	7.48	−4.39	−3.52		−1.57	−0.12	1.55	1.63	1.55	1.66	0.76	0.80	0.77	0.79	120.36	123.25	120.33	124.59	4.39	4.90	5.53	5.61	91.42	80.96	89.31	82.52	93.52	79.38
27	Yes	12.36	12.84	12.29	13.01	11.02	11.45	4.86	10.48	−2.07	−3.12	−1.52	−2.21	−4.94	−4.43	−5.65	−3.64	0.37	2.20	0.88	0.73	−1.01	0.40		−2.19	−2.57	1.17	1.19	1.17	1.20	0.68	0.70	0.68	0.68	103.43	102.72	103.06	103.75	2.78	3.26	3.21	3.38	104.13	111.46	101.84	110.60	106.42	112.32
28	No	13.25	13.46	9.20	14.74	10.29	10.46	7.40	12.28	−16.11	−14.14	−6.62	−14.29	−4.96	−5.49	−10.56	−5.95	0.45	1.11	−0.36	5.23	−2.92	−2.61		−2.63	2.52	1.17	1.21	1.22	1.20	0.61	0.62	0.62	0.63	114.22	116.72	121.53	114.99	3.45	3.81	3.43	4.21	92.96	101.17	94.38	102.09	91.54	100.25
29	Yes	18.78	19.14	13.97	16.36	10.62	11.94	10.48	10.49	−5.30	−4.01	−9.49	−6.74	−1.86	−2.68	−3.74	0.48	6.31	11.22	2.87	3.90	−0.51	5.28		−2.00	−2.07	1.34	1.07	1.37	1.07	0.79	0.70	0.79	0.69	102.58	92.22	104.53	92.19	3.98	3.01	4.24	3.20	78.16	90.27	90.99	84.82	98.00	95.73

All values represent an average value from 3 representative trials in each patient.

## References

[1] Barouk P. Technique, indications, and results of proximal medial gastrocnemius lengthening. Foot Ankle Clin. 2014;19(4):795-806. doi:10.1016/j.fcl.2014.08.012 25456723

[2] Barouk P Barouk LS. Clinical diagnosis of gastrocnemius tightness. Foot Ankle Clin. 2014;19(4):659-667. doi:10.1016/j.fcl.2014.08.004 25456715

[3] Carlson RE Fleming LL Hutton WC. The biomechanical relationship between the tendoachilles, plantar fascia and metatarsophalangeal joint dorsiflexion angle. Foot Ankle Int. 2000;21(1):18-25. doi:10.1177/107110070002100104 10710257

[4] Chimera NJ Castro M Davis I Manal K. The effect of isolated gastrocnemius contracture and gastrocnemius recession on lower extremity kinematics and kinetics during stance. Clin Biomech (Bristol, Avon). 2012;27(9):917-923. doi:10.1016/j.clinbiomech.2012.06.010 22819670

[5] Cimolin V Galli M Vimercati SL Albertini G. Use of the Gait Deviation Index for the assessment of gastrocnemius fascia lengthening in children with Cerebral Palsy. Res Dev Disabil. 2011;32(1):377-381. doi:10.1016/j.ridd.2010.10.017 21075594

[6] Correa KP Devetak GF Martello SK de Almeida JC Pauleto AC Manffra EF. Reliability and minimum detectable change of the Gait Deviation Index (GDI) in post-stroke patients. Gait Posture. 2017;53:29-34. doi:10.1016/j.gaitpost.2016.12.012 28073084

[7] Davis RB III Ounpuu S Tyburski D Gage JR. A gait analysis data collection and reduction technique. Hum Mov Sci. 1991;10(5):575-587.

[8] DiGiovanni CW Kuo R Tejwani N , et al. Isolated gastrocnemius tightness. J Bone Joint Surg Am. 2002;84(6):962-970. doi:10.2106/00004623-200206000-00010 12063330

[9] Gianakos A Yasui Y Murawski CD Kennedy JG. Effects of gastrocnemius recession on ankle motion, strength, and functional outcomes: a systematic review and national healthcare database analysis. Knee Surg Sports Traumatol Arthrosc. 2016;24(4):1355-1364. doi:10.1007/s00167-015-3939-3 26685692

[10] Guzik A Drużbicki M. Application of the Gait Deviation Index in the analysis of post-stroke hemiparetic gait. J Biomech. 2020;99:109575. doi:10.1016/j.jbiomech.2019.109575 31870656

[11] Hoefnagels EM Weerheijm L Witteveen AG Louwerens JK Keijsers N. The effect of lengthening the gastrocnemius muscle in chronic therapy resistant plantar fasciitis. Foot Ankle Surg. 2021;27(5):543-549. doi:10.1016/j.fas.2020.07.003 32773360

[12] Holtmann JA Südkamp NP Schmal H Mehlhorn AT. Gastrocnemius recession leads to increased ankle motion and improved patient satisfaction after 2 years of follow-up. J Foot Ankle Surg. 2017;56(3):589-593. doi:10.1053/j.jfas.2017.01.037 28476392

[13] Horsch A Petzinger L Deisenhofer J , et al. The impact of operative correction of equinus in cerebral palsy on gait patterns. Foot Ankle Int. 2024;45(2):130-140. doi:10.1177/10711007231217273 38156624

[14] Kark L Vickers D McIntosh A Simmons A. Use of gait summary measures with lower limb amputees. Gait Posture. 2012;35(2):238-243. doi:10.1016/j.gaitpost.2011.09.013 22000790

[15] Kung SM Fink PW Legg SJ Ali A Shultz SP. Age-dependent variability in spatiotemporal gait parameters and the walk-to-run transition. Hum Mov Sci. 2019;66:600-606. doi:10.1016/j.humov.2019.06.012 31277034

[16] Ma N Sclavos N Passmore E Thomason P Graham K Rutz E. Three-dimensional gait analysis in children undergoing gastrocsoleus lengthening for equinus secondary to cerebral palsy. Medicina (Kaunas). 2021;57(2):98. doi:10.3390/medicina57020098 33499373 PMC7911110

[17] Maanum G Jahnsen R Stanghelle JK Sandvik L Larsen KL Keller A. Face and construct validity of the Gait Deviation Index in adults with spastic cerebral palsy. J Rehabil Med. 2012;44(3):272-275. doi:10.2340/16501977-0930 22214985

[18] McGinley JL Baker R Wolfe R Morris ME. The reliability of three-dimensional kinematic gait measurements: a systematic review. Gait Posture. 2009;29(3):360-369. doi:10.1016/j.gaitpost.2008.09.003 19013070

[19] Molund M Husebye EE Hellesnes J Nilsen F Hvaal K. Proximal medial gastrocnemius recession and stretching versus stretching as treatment of chronic plantar heel pain. Foot Ankle Int. 2018;39(12):1423-1431. doi:10.1177/1071100718794659 30132688

[20] Molund M Husebye EE Nilsen F Hellesnes J Berdal G Hvaal KH. Validation of a new device for measuring isolated gastrocnemius contracture and evaluation of the reliability of the Silfverskiöld test. Foot Ankle Int. 2018;39(8):960-965. doi:10.1177/1071100718770386 29676167

[21] Monteagudo M de Albornoz PM Gutierrez B Tabuenca J Álvarez I. Plantar fasciopathy: a current concepts review. EFORT Open Rev. 2018;3(8):485-493. doi:10.1302/2058-5241.3.170080 30237906 PMC6134886

[22] Patel A DiGiovanni B. Association between plantar fasciitis and isolated contracture of the gastrocnemius. Foot Ankle Int. 2011;32(1):5-8. doi:10.3113/fai.2011.0005 21288428

[23] Pickin CC Elmajee M Aljawadi A Fathalla I Pillai A. Gastrocnemius recession in recalcitrant plantar fasciitis: a systematic review. J Foot Ankle Surg. 2022;61(2):396-400. doi:10.1053/j.jfas.2021.10.029 34838458

[24] Riddle DL Pulisic M Pidcoe P Johnson RE. Risk factors for Plantar fasciitis: a matched case-control study. J Bone Joint Surg Am. 2003;85(5):872-877. doi:10.2106/00004623-200305000-00015 12728038

[25] Riiser MO Husebye EE Hellesnes J Molund M. Outcomes after proximal medial gastrocnemius recession and stretching vs stretching as treatment of chronic plantar fasciitis at 6-year follow-up. Foot Ankle Int. 2024;45(1):1-9. doi:10.1177/10711007231205559 37902240 PMC10822063

[26] Røislien J Skare Ø Gustavsen M Broch NL Rennie L Opheim A. Simultaneous estimation of effects of gender, age and walking speed on kinematic gait data. Gait Posture. 2009;30(4):441-445. doi:10.1016/j.gaitpost.2009.07.002 19665379

[27] Rong K Li XC Ge WT Xu Y Xu XY. Comparison of the efficacy of three isolated gastrocnemius recession procedures in a cadaveric model of gastrocnemius tightness. Int Orthop. 2016;40(2):417-423. doi:10.1007/s00264-015-2860-1 26156718

[28] Schwartz MH Rozumalski A. The Gait Deviation Index: a new comprehensive index of gait pathology. Gait Posture. 2008;28(3):351-357. doi:10.1016/j.gaitpost.2008.05.001 18565753

[29] Sloot LH Malheiros S Truijen S , et al. Decline in gait propulsion in older adults over age decades. Gait Posture. 2021;90:475-482. doi:10.1016/j.gaitpost.2021.09.166 34619614

[30] Slullitel GA Martinez de Albornoz P Oller Boix A Rey Cañas R Vazquez Vidosa J Monteagudo de la Rosa M. Proximal medial gastrocnemius recession for recalcitrant plantar fasciitis. Foot Ankle Int. 2024;45(8):833-838. doi:10.1177/10711007241242792 38715313

[31] Sussman MD Gage JR Schwartz MH Koop SE Novacheck TF (eds) : The identification and treatment of gait problems in cerebral palsy. J Child Orthop. 2010;4(2):177-178.

[32] Wang Y Qi Y Ma B , et al. Three-dimensional gait analysis of orthopaedic common foot and ankle joint diseases. Front Bioeng Biotechnol. 2024;12:1303035. doi:10.3389/fbioe.2024.1303035 38456008 PMC10919227

